# Clinical, radiographical and histological evaluation of alveolar ridge preservation with an autogenous tooth derived particulate graft in EDS class 3–4 defects

**DOI:** 10.1186/s12903-021-01429-y

**Published:** 2021-02-11

**Authors:** Zsombor Radoczy-Drajko, Peter Windisch, Eszter Svidro, Peter Tajti, Balint Molnar, Gabor Gerber

**Affiliations:** 1 Department of Periodontology, Semmelweis University, Szentkirályi street 47., Budapest, 1088 Hungary; 2 Scientific Students’ Associations Student, Semmelweis University, Budapest, Hungary; 3 Department of Anatomy, Histology and Embriology, Semmelweis University, Budapest, Hungary

## Abstract

**Background:**

The shrinkage of alveolar bone dimensions after tooth extraction is a well-known issue. This clinical phenomenon poses a challenge for clinicians aiming at implant-prosthetic treatment. BonMaker^®^ ATB is a novel autogenous bone grafting material, produced by the mechanical and chemical processing of natural teeth. This pilot case report aims at providing a clinical, radiographical, and histological evaluation of the safety and efficacy of Bonmaker ATB powder in the treatment of EDS class 3–4 postextraction sockets with alveolar ridge preservation.

**Methods:**

A total of 9 teeth were extracted from 5 patients. The extracted teeth were prepared immediately with the Bonmaker^®^ device. The extraction sockets were filled up with ATB powder. Six months after extraction, standardized intraoral x-rays and CBCT scans were performed. Re-entry was performed under local anaesthesia. Core biopsies were harvested for histological analysis and implants were placed.

**Results:**

Horizontal alveolar dimension loss occurred, even though ARP was performed, but the horizontal shrinkage was moderate. Vertical dimensions did not show loss of volume, but increased defect fill. Core biopsies showed ATB particles surrounded by newly formed bone and connective tissue. According to histomorphometric analysis, the harvested samples contained 56% of newly formed bone on average, and only a mean of 7% of non-remodelled ATB material was observed.

**Conclusion:**

The preliminary clinical, radiographical, and histological results of Bonmaker^®^ autogenous tooth graft therapy indicate that ATB may be safely and successfully used as a grafting material for ARP. Optimal graft incorporation and histologically proven effective remodelling, as well as uneventful wound healing support the clinical application of ATB to minimize post-extraction hard tissue loss. Further research is needed to exploit the full potential of ATB and to evaluate the long-term peri-implant hard and soft tissue stability of ATB-treated post-extraction sites.

## Background

Shrinkage of alveolar bone dimensions after tooth extraction is a well-known issue [1,2]. The dimensional changes of the alveolar ridge take place in the first eight weeks after tooth extraction; hard tissue volume loss is more profound in the buccal aspect of the alveolar process [1,3]. In addition, not only horizontal but also vertical loss can be expected in both hard tissue and soft tissue volume [4]. This clinical phenomenon poses a challenge for clinicians aiming at implant-prosthetic treatment with favourable aesthetics and long-term success. Alveolar ridge preservation (ARP) procedures aim at facilitating the healing of extraction sites scheduled for implant treatment in cases where immediate implant placement is contraindicated. The majority of these procedures are based on the application of space-maintaining osteoconductive xenogeneic, allogenic, or synthetic bone substitutes alone or in combination with resorbable and non-resorbable barrier membranes [5].

Human dentin and enamel histologically consist of 55% inorganic structure (mostly hydroxyapatite) and 45% organic content [6]. The organic part contains bone morphogenic proteins (BMP) and mostly type I collagen. The type I collagen content makes up about 90% of the dentin extracellular matrix [7]. The organic and inorganic structure of the human tooth resembles those of human bones. Containing organic and inorganic parts, human tooth particles have osteoinductive and osteoconductive capacities, therefore teeth can be used as a material for ARP purposes [8,9]. Based on literature data on grafting approaches, in which xenogeneic grafting materials where used alone for alveolar ridge preservation purposes, the human tooth derived Bonmaker^®^ auto-tooth graft (ATB) may be a successful material for ARP [5,9,13]. BonMaker^®^ ATB is a novel autogenous bone grafting material, produced by mechanical and chemical processing of extracted natural teeth. Thus, a novel ARP approach without the need for xenogeneic, allogenic or synthetic bone substitutes may be introduced, utilizing the highly resorbable and biocompatible ATB material as a space maintaining device.

In addition to clinical concepts and materials used for ARP, not only the reconstructive technique affects the healing of the alveolar ridge, but so does the atraumatic extraction by preserving alveolar bone walls [10] The loss of hard tissue volume is even more intense in postextraction alveolar sockets, where buccal or oral dehiscences are present prior to extraction. The surgical reconstruction of such sockets demonstrating a non-contained defect configuration is very challenging in terms of maintaining the original dimensions, according to the Extraction Defect Sounding (EDS) classification [11].

The present pilot case report aims to provide a clinical, radiographical and histological evaluation of the safety and efficacy of Bonmaker ATB powder in the treatment of EDS class 3–4 postextraction sockets.

## Materials and methods

**Fig. 1:**
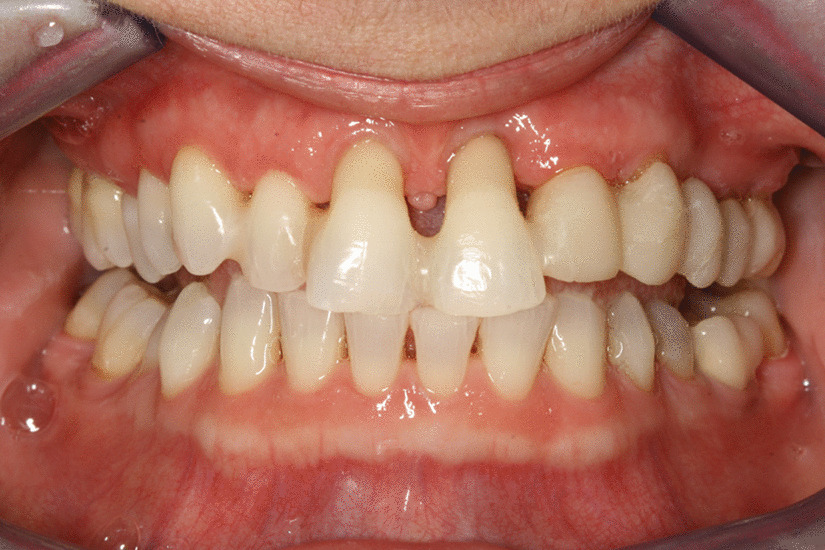
Baseline clinical view: middle incisors with periodontally hopeless prognosis

**Fig. 2:**
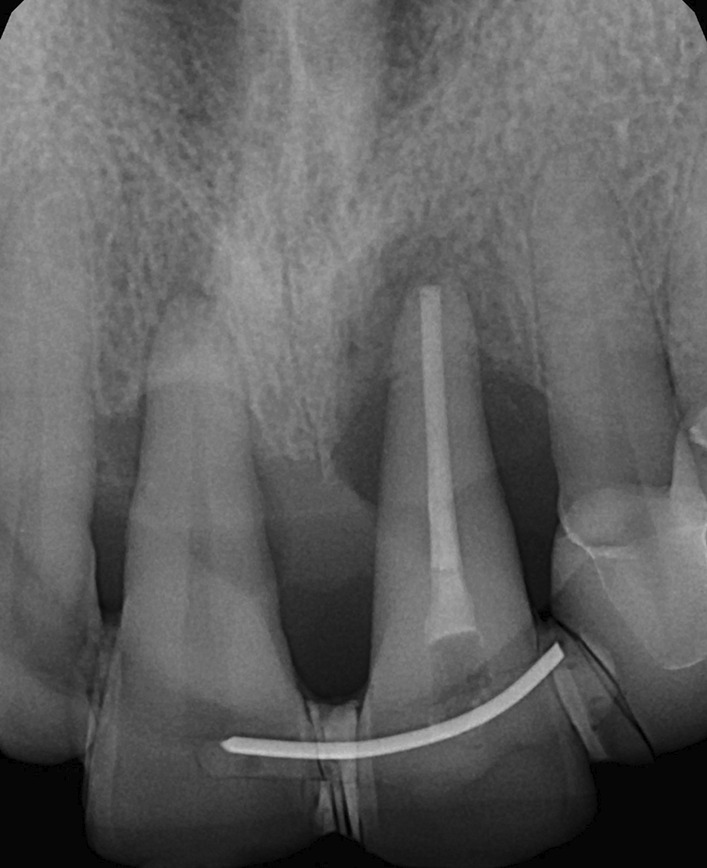
Baseline intraoral X-ray

**Fig. 3:**
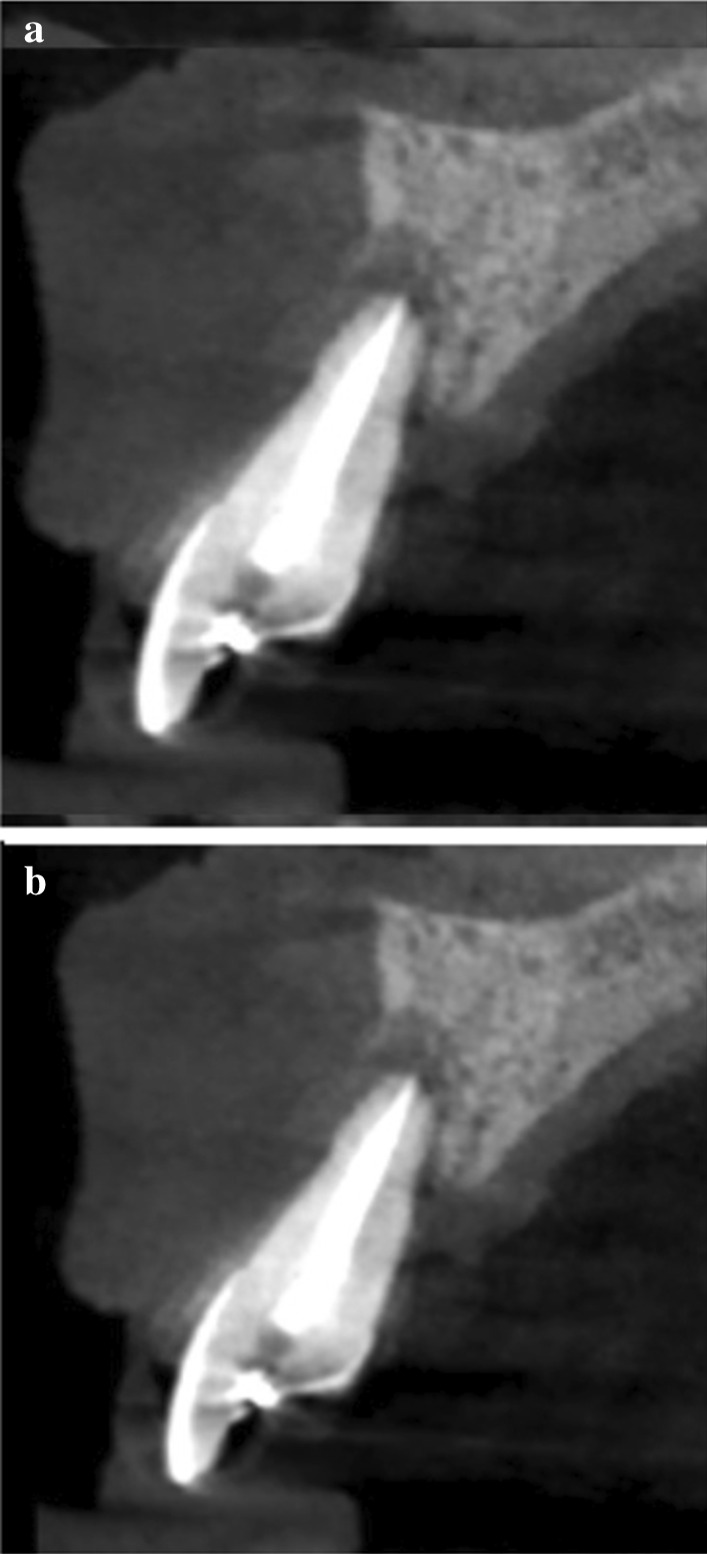
Baseline CBCT scan of upper middle incisors, (**a**) upper right first incisor, (**b**) upper left first incisor

5 patients aged 18–70 with good general health and proper individual oral hygiene (confirmed by full mouth plaque and bleeding scores below 20%) underwent initial periodontal treatment prior to tooth extraction [12], followed by ARP utilizing ATB and a free gingival graft (FGG) at the Department of Periodontology, Semmelweis University, Budapest, Hungary between January, 2017 and January, 2019. The study was approved by the Semmelweis University Regional and Institutional Committee of Science and Research Ethics (Approval Document Number 54781–2/2016/EKU, 26.10.2016.). The patients were treated in full accordance with ethical principles, including the World Medical Association Declaration of Helsinki (version 2008). Surgical interventions were undertaken with the understanding and written consent of each subject. The exclusion criteria were: major relevant clinical diseases, untreated periodontal disease, systemic use of steroids, current or previous intravenous bisphosphonate treatment, chronic or acute periapical infection at the operation site, and previous GBR/GTR treatment at the extraction site. Each patient presented at least one single rooted tooth scheduled for extraction (Fig. 1). All enrolled patients were examined by two independent periodontists to assure that the teeth scheduled for extraction were hopeless. Standardized intraoral X-rays (Fig. 2) and CBCT scans (Fig. 3a, b) were taken preoperatively at the sites of interest. Hard tissue dimensions and alveolar socket dimensions were measured in the CBCT images preoperatively.

A total of 9 hopeless teeth (proven by two independent clinicians) were extracted under local anaesthesia with articaine 4% and epinephrine 1:100.000 (Ultracain, Sanofi Aventis, Paris, France). The extractions were carried out with the gentle application of a forceps and an elevator, if needed. Extraction sockets were debrided utilising a flapless approach with Lucas-instruments and scalpels. The extent of the buccal dehiscence and EDS classification were confirmed by direct clinical measurements using PCP-UNC 15 periodontal probes. Patients presenting EDS Class 3–4 defects with a minimum of 3 mm vertical buccal bone dehiscence were enrolled to the study. The extracted teeth were prepared immediately after removal. The process was performed using the Bonmaker^®^ device, following the developers’ instructions. First, the outer surfaces of the teeth were cleaned with a diamond-coated bur. After that, resin- and root canal filling materials, remaining pulp tissues and any restorations were removed using diamond-coated burs. The cleaned teeth were prepared with the Bonmaker^®^ tooth grinder; crowns and roots were also crushed, with both containing dentin as well as enamel. After grinding, particulate tooth material underwent a 3-step disinfection and preparation process via proprietary A, B, C solutions within the Bonmaker^®^ device, according to the manufacturer’s protocol, resulting in ready-to-use ATB. During the preparation and disinfection procedure, which usually took 30–35 min (10–15 min preparation, 20 min disinfection), soft tissue grafts were harvested. Between two patients, the device underwent a disinfection cycle according to the manufacturers’ instructions. All the parts, which came in contact with the extracted teeth, underwent autoclave sterilization to avoid cross-infection.

**Fig. 4:**
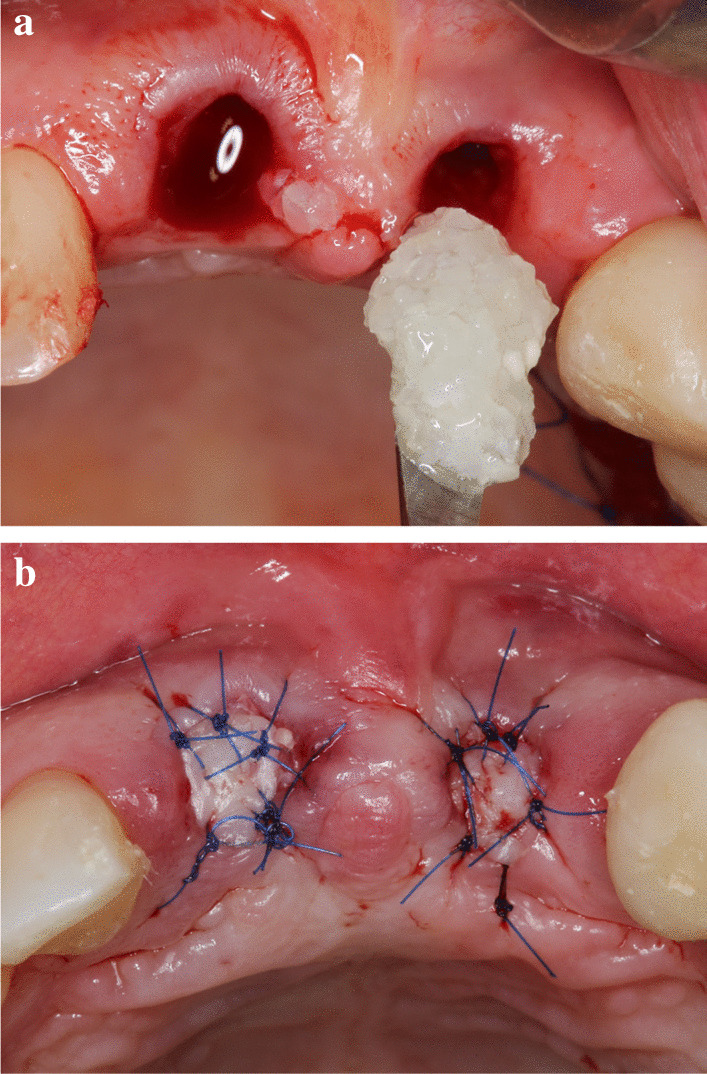
(**a**) Alveolar ridge preservation was carried out with the disinfected auto-tooth bone graft (BonMaker^®^, Korea Dental Solutions Co. Ltd., South Korea), (**b**) Post extraction sockets covered with FGG transplants

Subsequently, the extraction sockets were filled up with ATB powder (Fig. 4a) (particle size: 425 µm–1500 µm). To ensure optimal soft tissue coverage and the undisturbed healing of the grafted sites over 6 months of healing, free gingival grafts (FGG) were harvested from the palate or the maxillary tuberosity and were adapted to the extraction site by 6/0 non-resorbable monofilament sutures (Dafilon, Braun B Melsungen, Tuttlingen, Germany) (Fig. 4b).

**Fig. 5.**
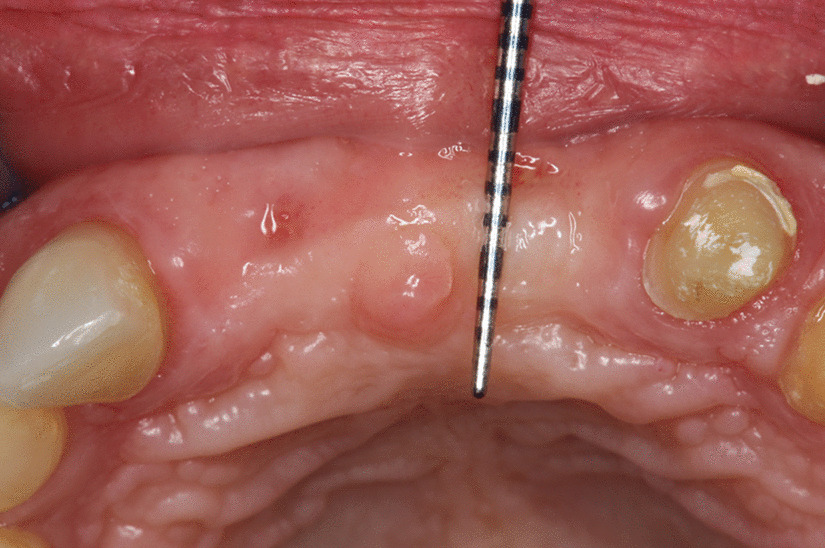
6-month postoperative view, before re-entry

**Fig. 6.**
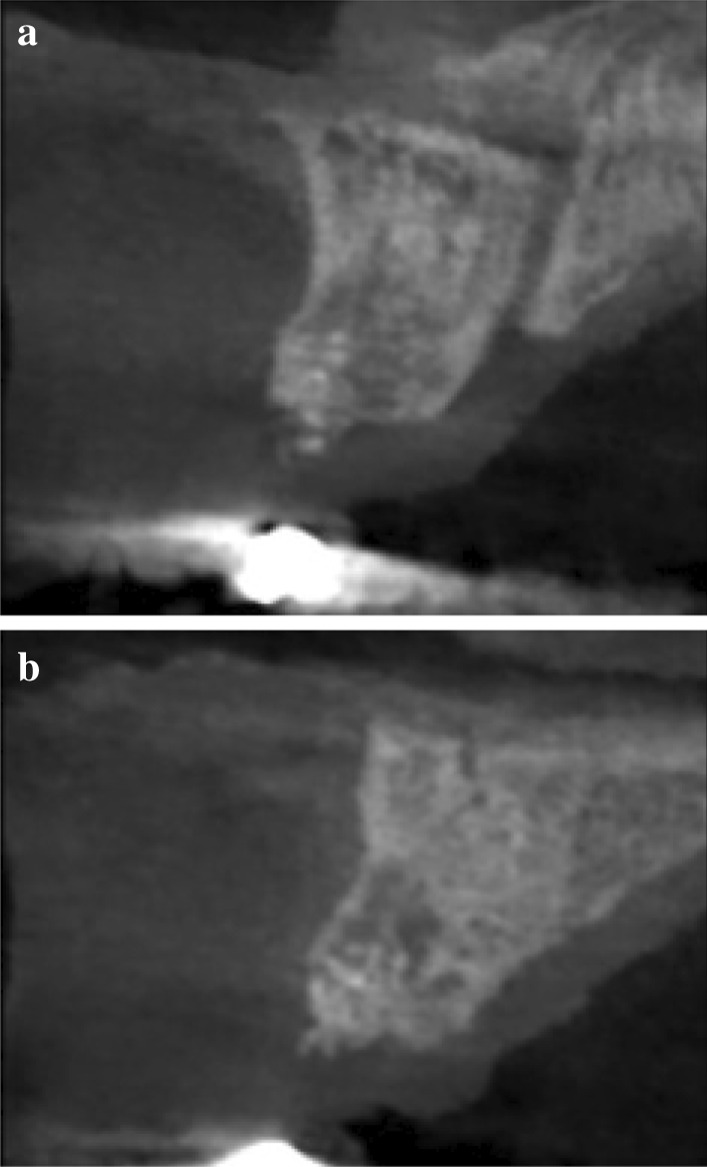
6-month postoperative CBCT scan before re-entry, (**a**) upper right first incisor, (**b**) upper left first incisor

**Fig. 7:**
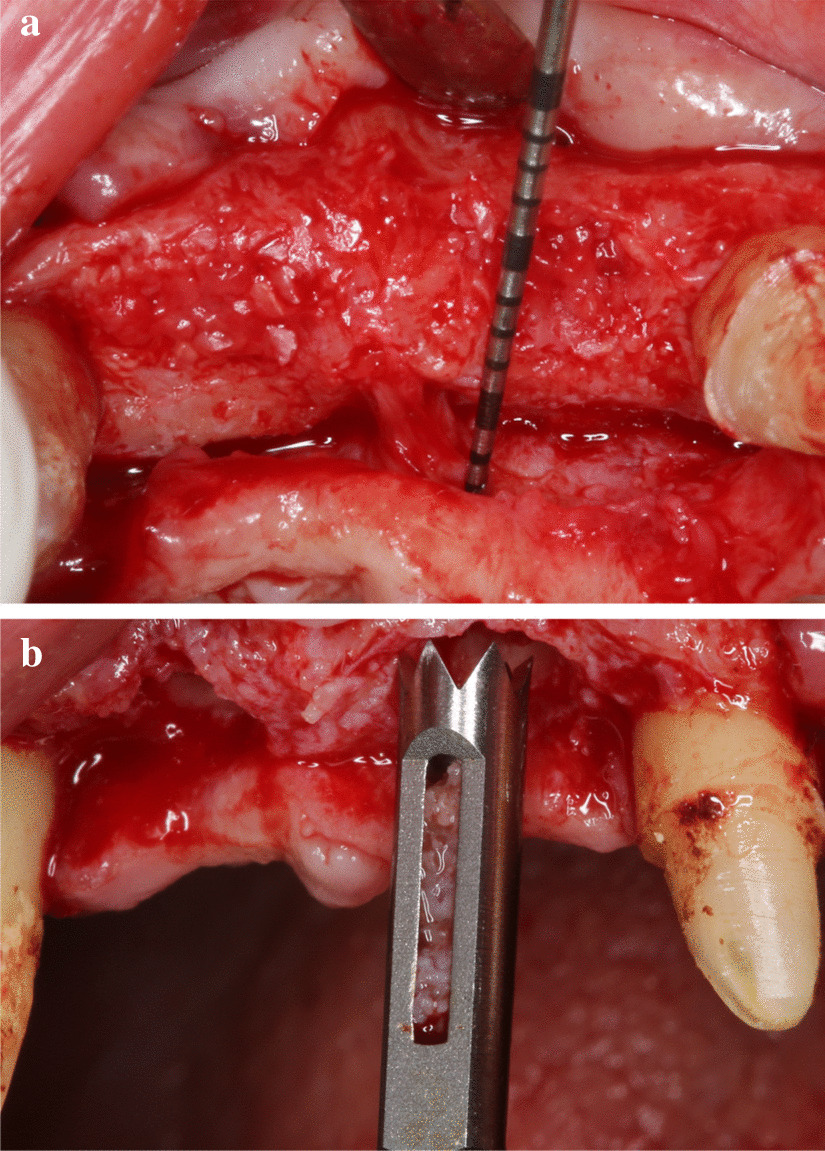
(**a**) Intraoperative picture before histological sample harvesting, (**b**) Histological sample harvesting with trephine burs

**Fig. 8:**
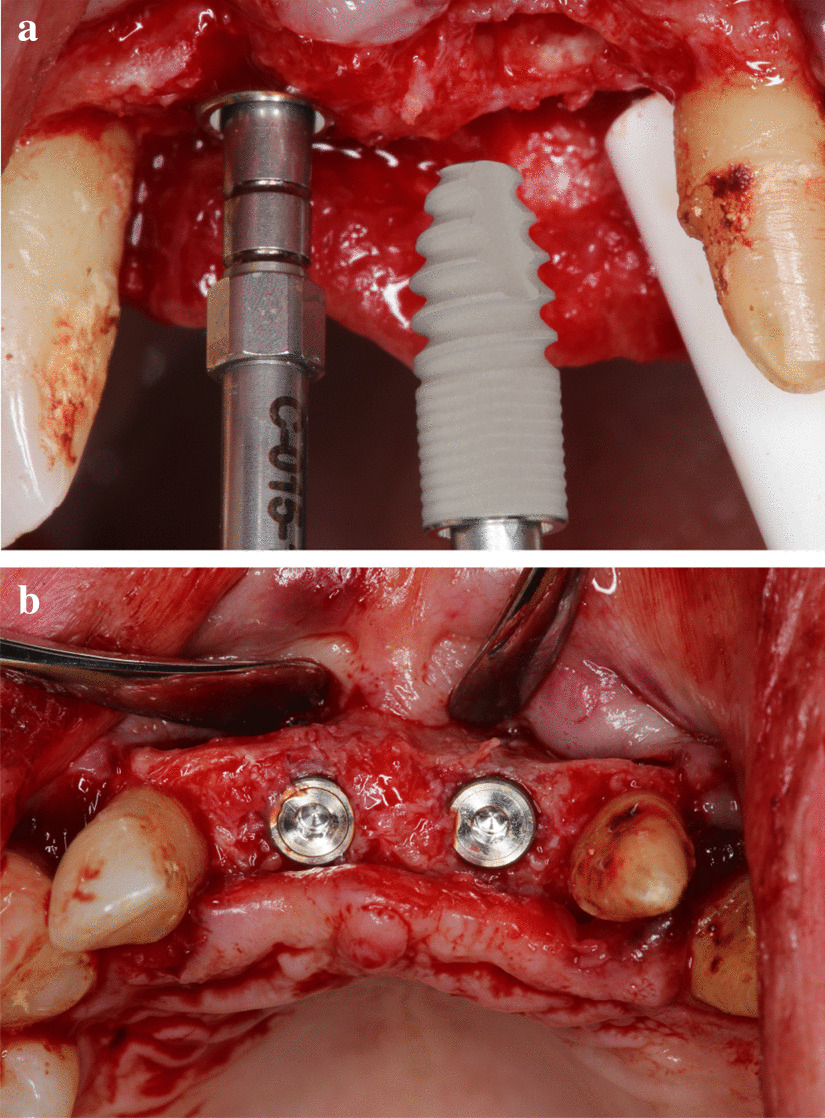
(**a**) Implant placement (ICX, Medentis Medical GmbH, Germany) after the histological sample harvesting, (**b**) Implants in final position

Standardized intraoral X-rays were taken before and immediately after surgery at the sites of interest. Suture removal was performed 14 after the surgery. Qualitative soft tissue assessment was performed 1, 3, and 6 months postoperatively (Fig. 5). Six months after extraction, standardized intraoral x-rays and CBCT scans (Fig. 6a, b) were taken at the sites of interest prior to the re-entry procedure for a radiographic evaluation of hard tissue changes. Re-entry was performed under local anaesthesia (Fig. 7a). The amount of newly formed hard tissue was assessed prior to re-entry by CBCT evaluation. Core biopsies were harvested (Fig. 7b) for histological analysis at implant osteotomy sites using 2.6 mm inner/3.6 mm outer diameter trephine burs (Komet Dental, Lemgo, Germany) prior to implant placement in 10 mm depth in the planned implant axis. Subsequently, implants were placed (ICX, Medentis Medical GmbH, Germany) (Fig. 8a, b) in the position of the core biopsy harvesting, following additional implant osteotomy by proprietary drills. If any buccal bone dehiscenses were presented at implant surfaces after fixture placement, simultaneous guided bone regeneration (GBR) was performed using the combination of autogenous bone and xenogeneic grafting material (cerabone^®^, botiss, Germany). The composite graft was covered with resorbable membranes (Jason^®^, botiss, Germany) and fixed to the periosteum with titanium pins or sutures.

Core biopsy samples were decalcinated, fixed in formalin, embedded in paraffin. 10 ųm wide sections were prepared. Subsequently, haematoxylin and eosin (H&E) staining was performed, blind quantitative histological analysis was carried out by three trained specialists using the Neurolucida^®^ (MBF Bioscience, Williston, USA) software individually adapted for histomorphometry.

**Fig. 9:**
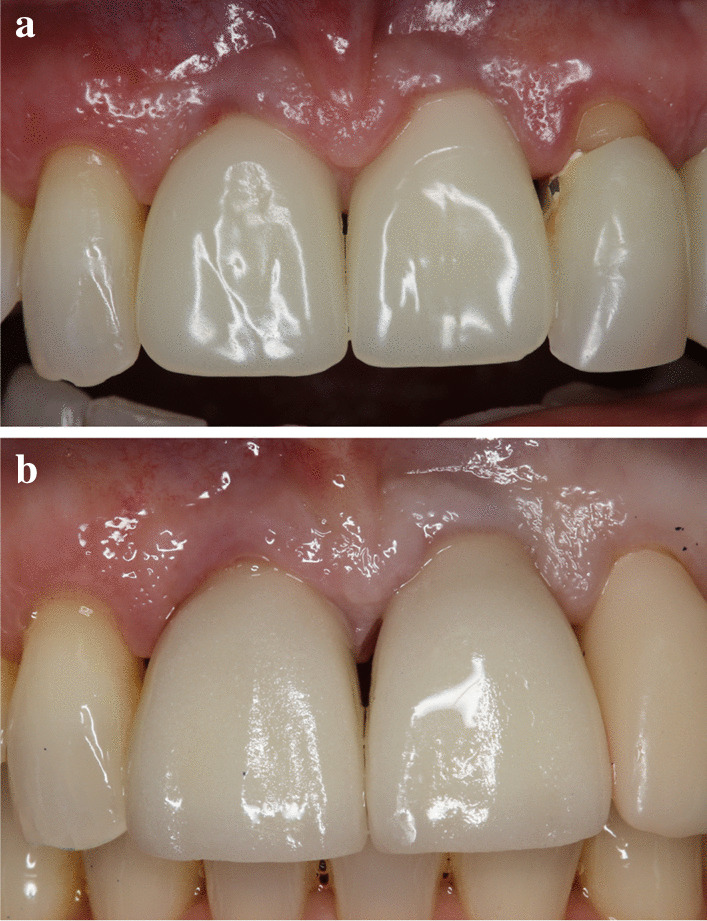
(**a**) Temporary restorations, (**b**) Final restorations

**Fig. 10:**
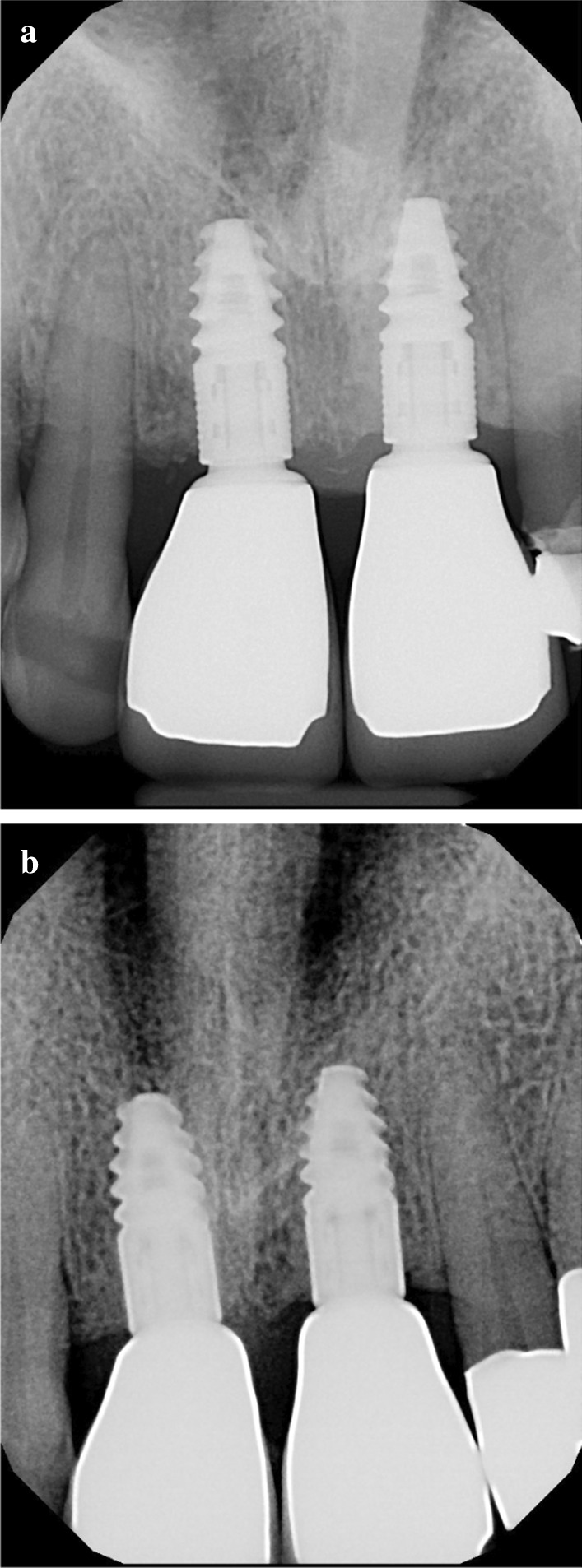
(**a**) Intraoral X-ray at delivery of final restorations, (**b**) 12 months after delivery

6 months after implant placement, the implants were uncovered, emergence profiles were shaped with temporary restorations (Fig. 9a), 1 month later, final restorations were delivered (Fig. 9b). Intraoral x-rays were taken with the final restorations in place (Fig. 10a), and 12 months later, no signs of negative bone remodelling were seen (Fig. 10b).

## Results

Initial wound healing was uneventful, with no signs of graft exposure over the complete healing period of 6 months. The quality of newly formed keratinized tissues was favourable at 1, 3 and 6 months, allowing for optimal aesthetic implant rehabilitation in all cases. At re-entry, opposed to the common behaviour of xenogeneic grafts, ATB augmented sites did not exhibit any signs of graft connective tissue encapsulation. As a result, crestal bone maintenance was ensured. Implant placement was possible at all 9 sites treated with ATB, all implants were embedded in vital hard tissue, threads were fully covered. In 7 cases out of 9, minor simultaneous contour augmentation was carried out due to the lack of buccal tissue volume. In one case, implant placement was combined with sinus floor elevation to augment a pneumatized maxillary sinus.

Preoperative and 6-month postoperative CBCT scans were taken for radiographic analysis (Figs. 3a, b, Fig. 6a, b). The midline axes of the alveolar sockets were defined manually in mid-buccal oro-vestibular cross-sections. The accurate comparison of pre- and postoperative data sets was possible by selecting standardized reference points using adjacent anatomical landmarks at the base of the alveolar process, along the midline axis. Horizontal measurements were performed at 3 levels: at the crest of the alveolar socket, at 2 mm below the crest, and at 4 mm below the crest. Vertical measurements were performed from the crest of the alveolar socket to the standardized basal reference point.

Mean baseline and 6 months horizontal and vertical socket dimension measurements are shown in Table 1.

**Table 1:** Mean horizontal vertical alveolar socket dimensions in mid-buccal oro-vestibular cross-sections of baseline and 6-month CBCT scans

Alveolar dimensions	Baseline	6 months
Width at crest	8.14 ± 1.82 mm	6.74 ± 1.11 mm
Width 2 mm below crest	8.64 ± 1.82 mm	7.45 ± 0.92 mm
Width 4 mm below crest	9.15 ± 1.47 mm	8.09 ± 0.78 mm
Height from reference point	12.35 ± 3.44 mm	14.61 ± 5.74 mm

As indicated in Table 1, horizontal alveolar dimension loss occurred, regardless of ARP. Nevertheless, the moderate horizontal shrinkage did not preclude implant placement in any of the cases. Horizontal alveolar dimension loss was 20.7% at the most coronal aspect of the alveoli, 15.9% at 2 mm below the coronal measurement line, and 13.1% at 4 mm below the coronal measurement line. Unlike horizontal changes, vertical dimensions did not show loss of volume, but increased defect fill instead. Mean vertical dimension gain at 6 months was 18.3% compared to baseline. Supracrestal ATB particles were maintained and were visibly attached to newly formed subcrestal hard tissues at re-entry surgery. Nevertheless, inferior structural integrity was observed compared to the subcrestal area. From the clinician’s perspective, the consistency of the ATB-preserved sites was close to D3-D4 bone during implant osteotomy. In cases with a thin mucosal biotype slight initial negative bone remodelling was observed following implant uncovery (Fig. 10a). Nevertheless, crestal bone stability was maintained after the establishment of the biological width, as confirmed by 12 months follow-up X-rays (Fig. 10b).

**Fig. 11:**
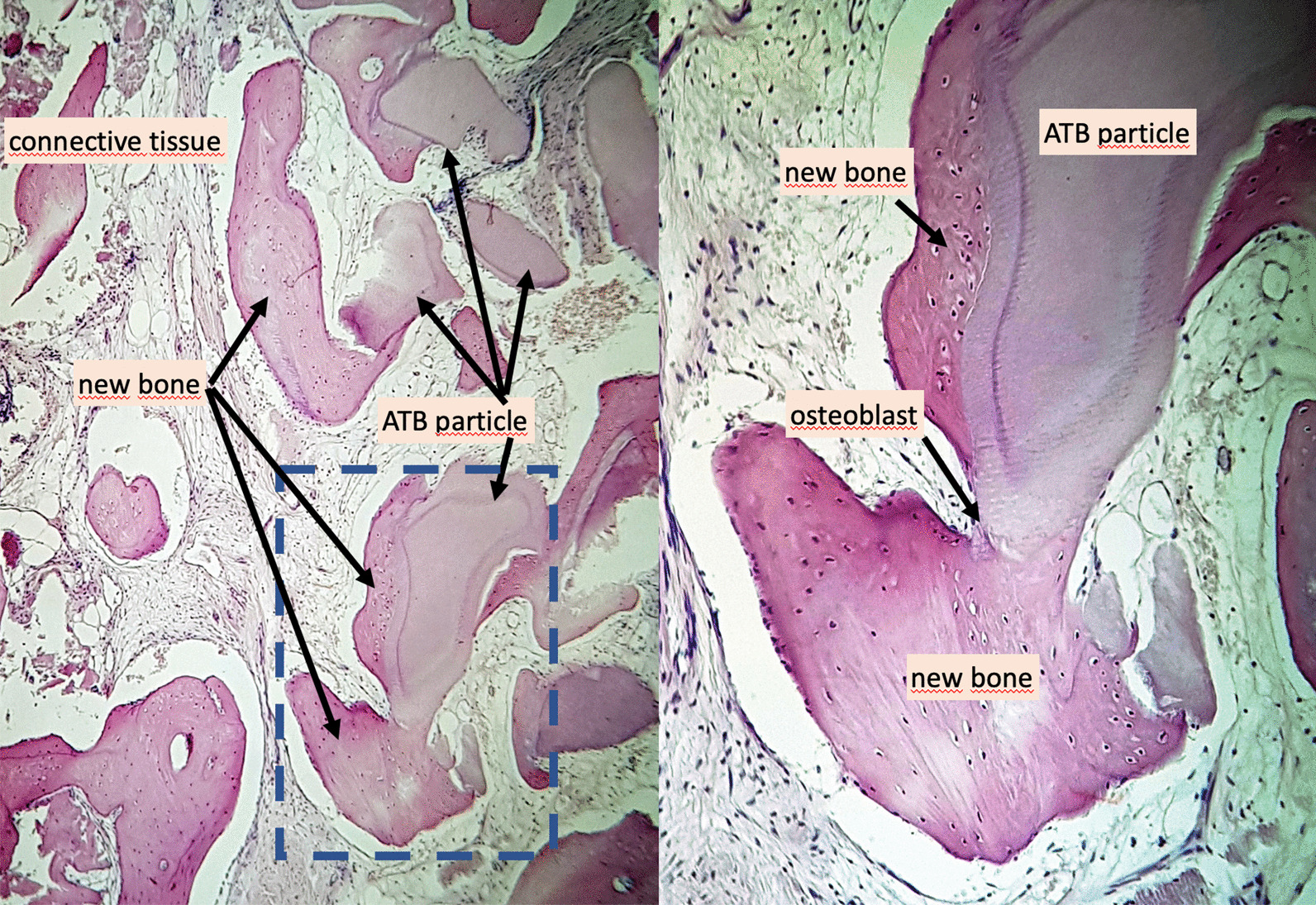
Histological overview, × 100 magnification, Haematoxylin–eosin staining

**Fig. 12:**
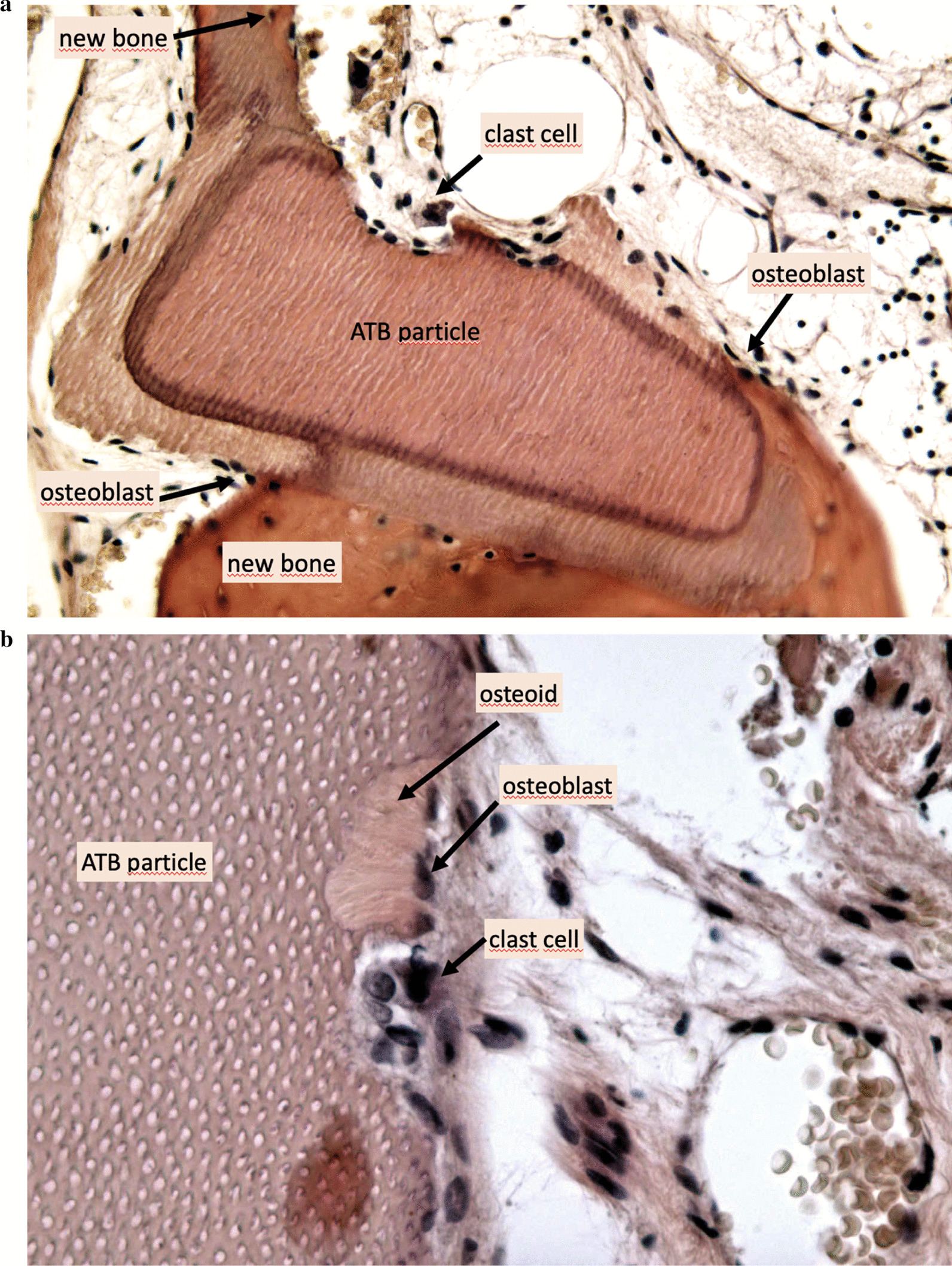
(**a**) Histological view, × 200 magnification, Haematoxylin–eosin staining, (**b**) Histological view, × 400 magnification, Haematoxylin–eosin staining

The overview sections of the core biopsies (Fig. 11) show ATB particles surrounded by newly formed bone and connective tissue. The resorption lacunae with large, multinucleated osteoclasts and the remodeling sites with osteoblasts adjacent to osteoid tissue are clearly visible (Fig. 12b). Osteocytes can be seen in the newly formed bone with close contact to the ATB particles (Fig. 12a).

According to histomorphometric analysis, the harvested samples contained 56% of newly formed bone on average, and only a mean of 7% of non-remodelled ATB material was observed. An average of 37% connective tissue was found in the biopsies. The results of the histomorphometric analysis are shown in Table 2.

**Table 2:** Histological evaluation of the core biopsies with the Neurolucida^®^ software (preoperative tooth positions in FDI tooth numbering)

	Bone proportion (%)	ATB material proportion (%)	Connective tissue proportion (%)
Sample 1 (11)	40	8	52
Sample 2 (21)	36	12	52
Sample 3 (27)	51	7	42
Sample 4 (23)	52	0	48
Sample 5 (24)	56	0	44
Sample 6 (13)	45	21	34
Sample 7 (21)	73	10	17
Sample 8 (22)	68	7	25
Sample 9 (32)	80	2	18
Mean values	56	7	37

## Discussion

In our recent study, we successfully demonstrated the safety and efficacy of ATB powder in ARP procedures. We are the first to report clinical, radiographical, and histological data demonstrating favourable defect fills of EDS-class 3–4 postextraction sockets utilizing an autogenous tooth-derived bone grafting material.

Bone substitute materials may be used alone for ARP: deproteinized bovine bone material [13], demineralized freeze-dried bone allograft [14], hydroxyapatite crystals [15], bioglass [16], polylactide and polyglycolide sponge [17] have been proposed as osteoconductive scaffolds. Several types of membranes have been suggested for alveolar ridge preservation purposes: extended polytetrafluoroethylene membrane [18], bioabsorbable membrane from lactide and glycolide polimers [19], dense polytetrafluoroethylene [20]. In addition, membranes may be combined with bone grafting materials as well: dense polytetrafluoroethylene membrane and grafting material [21], collagen membrane and freeze-dried bone graft [22], xenogenic bone substitutes combined with a collagen barrier membrane [23,24]. Although there are many surgical methods that focus on maintaining hard tissue volume, none of them can be defined as ‘gold standard’, since post-extraction ridge resorption cannot be eliminated totally according to literature [5,25]. The Bonmaker^®^ device is feasible for grinding, disinfecting, and preparing extracted teeth to obtain ATB, a ready-to use particulate grafting material. ATB processing allows for producing both particulate and block grafts. Based on previous literature data describing the clinical handling and application of biomaterials in ARP procedures, particulate ATB powder and ATB blocks may act as a resorbable scaffold and space maintaining device to facilitate the healing of acute and chronic alveolar defects [6,8].

During our current study, the ATB powder preparation procedure took approximately 30 min, including pre-cleaning with diamond-coated burs and removal of restorations. While the device was in operation, the thorough removal of inflammatory tissues from the alveoli, as well as harvesting FGG’s from patients’ hard palate could be performed in a time-efficient manner. ATB exhibited excellent handling properties. After the preparation, the graft material became wet and sticky, which made it comfortable to use. To ensure the compaction of the grafting material inside the alveolar socket, osteotomes were applied. Wound healing was uneventful, major adverse events were not observed in any of the treated cases. In two out of the nine extraction sites, minor adverse events occurred: the epithelial layer of the FGG partially necrotized and had to be removed after one week. Even in these cases, after the removal of the necrotized epithelium, the grafting material was fully covered with connective tissue, indicating that FGG would facilitate wound healing by protecting the underlying grafted area. Secondary intention wound healing resulted in new keratinized tissue formation and complete graft coverage at 3 weeks postoperatively. 6 months after ARP, re-entry procedure was performed in order to place implants and harvest core biopsies from the preserved sites. According to Schropp et al. 2003, in the first 12 months after tooth extraction, on average less than 1 mm vertical remodelling occurs (+ 0.4 mm palatally, − 0.8 mm buccally). Due to ridge atrophy, as much as 50% of the original width is lost (6.1 mm on average) during spontaneous healing. In our study, similarly no vertical shrinkage was observed. Moderate loss of horizontal dimension of the alveolar crest was detectable at all sites, however, the shrinkage was minimal (15% on average), considering the fact that all the sites were previously qualified as EDS class 3–4 defects, exhibiting compromised healing capacity, representing a biological challenge for clinicians in terms of complete defect reconstruction. In some cases, supracrestal graft particles were maintained until re-entry, nevertheless, this cannot be considered as vertical socket augmentation, since supracrestal ATB parts were not always as mature as the subcrestal proportion of the reconstructed area.

The placement of fixtures in native bone was possible after core biopsy harvesting in all cases. Nevertheless, to compensate for the horizontal dimension loss for aesthetically favourable outcomes, contour augmentation was carried out in the buccal aspect in 6 out of 9 implant sites. The osseointegration of the inserted implants was successful, implant-fixed partial dentures were delivered after implant uncovery.

According to a systematic review, histological analysis of alveolar ridge preservation procedures revealed inferior hard tissue quality compared to native bone following all currently known approaches [5]. Cardaropoli et al. performed socket preservation with bovine bone mineral combined with 10% collagen and found 26.6% of newly formed bone with 18.5% grafting material surrounded by 55% connective tissue [26]. Barone et al. carried our ARP with a corticocancellous porcine graft and a resorbable collagen membrane. After at least 7 months of healing, core biopsies were harvested from the extraction sites: histomorphometrically they found 35.5% bone, 29.2% grafting material and 36.6% connective tissue [27]. Artzi et al. used porcine-based grafting material for socket preservation, and after 9 months of healing, re-entry procedure was performed, core biopsies were harvested. During histomorphometrical evaluation, the authors found 46.3% average bone fraction, 30.8% grafting material, and 22.9% connective tissue part^28^. Comparing our results with literature data reporting on limited graft remodeling in newly formed hard tissues following ARP, the histological evaluation in our current study confirmed an excellent remodeling of the ATB material. As shown by the histomorphometric analysis, the mean value of newly formed bone was 56%, which was significantly higher compared to data reported in literature. Six samples out of nine yielded more than 50% of new bone, which indicated exceptional tissue quality. In the harvested samples, only 7% of non-remodelled ATB material was observed on average, which indicated a more rapid turnover of graft particles compared to literature data reporting on xenogeneic materials used in ARP. A mean of 37% connective tissue was found in the samples, this was in line with previous observations following ARP with particulate grafting materials. Histomorphometrical analysis was performed in the complete biopsy area. In further studies, it is necessary to evaluate the graft integration pattern differences along the apicocoronal axis of core biopsies.

Compared to literature data, the significant amount of newly formed bone and the low amount of non-remodelled graft particles yielded a more favorable quality of hard tissues, confirmed by direct assessment during re-entry. ATB was capable of osteoinduction and osteoconduction, and newly formed hard tissues resembled native bone structurally, both intraoperatively and histologically. Moreover, the immediate application of ATB as an autogenous grafting material was cost- and time-efficient for the patient. The application of ATB with FGG coverage proved to be an ARP technique, which may limit post-extraction alveolar bone loss and, at the same time, provide favorable hard- and soft tissue quality.

## Conclusions

The preliminary clinical, radiographical, and histological results of Bonmaker^®^ autogenous tooth graft therapy indicate that ATB may be safely and successfully used as a grafting material for ARP. Optimal graft incorporation and histologically proven effective remodelling, as well as uneventful wound healing support the clinical application of ATB to minimize post-extraction hard tissue loss. Further research is needed to exploit the full potential of ATB and to evaluate the long-term peri-implant hard and soft tissue stability of ATB-treated post-extraction sites.

## Data Availability

All data generated or analyzed during this study are included in this published article.
